# Is the neutrophil-to-lymphocyte "ratio"nale from head and neck to thyroidology for thyroidologists?: promise or passé?

**DOI:** 10.1590/1806-9282.20231617

**Published:** 2024-05-20

**Authors:** Ilker Sengul, Demet Sengul, Tugrul Kesicioglu, Esma Cinar, Dzemail Detanac, Anton Pelikán, Pavla Kudlova

**Affiliations:** 1 Giresun University, Faculty of Medicine, Division of Endocrine Surgery – Giresun, Turkey.; 2 Giresun University, Faculty of Medicine, Department of General Surgery – Giresun, Turkey.; 3 Giresun University, Faculty of Medicine, Department of Pathology – Giresun, Turkey.; 4 General Hospital Novi Pazar, Department of General Surgery – Novi Pazar, Serbia.; 5 University Hospital Ostrava, Department of General Surgery – Ostrava, Czechia.; 6 University of Ostrava, Faculty of Medicine, Department of Surgical Studies – Ostrava, Czechia.; 7 Tomas Bata University in Zlín, Faculty of Humanities, Department of Health Care Sciences – Zlín, Czechia.

Dear Editor,

We read with a great deal the research article entitled "Is the neutrophil-to-lymphocyte ratio a marker for differentiating between benign and malignant submandibular gland masses?" by Bora^1^. This research of high quality seems to demand portraying to evaluation of the effect of the neutrophil-to-lymphocyte ratio (N/L) on distinguishing benign from malignant masses in the submandibular triangle of the head and neck which has recently been published in the 69th volume of *Rev Assoc Med Bras* ^1^. The author stated that he retrospectively evaluated 48 cases who had undergone surgery for submandibular gland masses with the histopathology of sialolithiasis, sialadenitis, pleomorphic adenoma^2–4^, and malignant conditions. He deduced and emphasized that N/L, *per se*, can be used as a biomarker in submandibular gland masses and has prognostic significance in malignant masses. Moreover, he proposed that N/L can be utilized as a biomarker beyond fine needle aspiration cytology. Thereto, he highlighted N/L as being proven in distinguishing between benign and malignant nodules in patients^1^ with suspicious thyroid nodules^1,5–17^. Nevertheless, at least a minimal debate is still ongoing on an accurate decision on N/L whether it is a biomarker in discrimination of malignity or not. Exempligratia, we reported that N/L was not a convenient and favorable biomarker in forecasting thyroid malignancy^18^ in thyroidology^19–29^. Of note, N/L, particularly in the thyroid with head and neck providers, the growing spectrum of clinical management for challenging conditions remains, especially for thyroidologists and thyroid health as different peas in a pod. *Fide, sed cui vide.* As a matter of fact, this issue merits further investigation. We thank Bora^1^ for his valuable study.
